# Implementation and use of standardized outcome measures by physical therapists in Saudi Arabia: barriers, facilitators and perceptions

**DOI:** 10.1186/s12913-017-2693-2

**Published:** 2017-11-21

**Authors:** Tahani N. Al-Muqiren, Einas S. Al-Eisa, Ahmad H. Alghadir, Shahnawaz Anwer

**Affiliations:** 1 King Abdulaziz Medical City, Riyadh, Saudi Arabia; 2 Rehabilitation Research Chair, College of Applied Medical Sciences, King Saud University, Riyadh, Saudi Arabia; 3 Dr. D. Y. Patil College of Physiotherapy, Dr. D. Y. Patil Vidyapeeth, Pune, India

**Keywords:** Outcome, Standardized measure, Physical therapy, Rehabilitation, Barriers, Facilitators

## Abstract

**Background:**

The use of standardized outcome measures (SOMs) has been recommended in the physical therapy practice guidelines to improve the patient’s management and encourage the evidence based practice. However, the extent of the use of SOMs by physical therapists (PTs) in Saudi Arabia was not investigated. The present study aimed to (1) evaluate the extent of the use of SOMs by PTs in routine daily practice in Saudi Arabia; (2) explore the barriers, facilitators and perceptions in the use of SOMs during physical therapy services; (3) examine the relationship between facility settings and the PTs characteristics and the use of SOMs.

**Methods:**

The present study used an observational design. A survey based questionnaire used and distributed to 352 PTs who were working in Saudi Arabia and was commonly involved in the management of patients within different clinical settings, either private or public.

**Results:**

One-hundred-eighty participants completed the questionnaires (response rate of 51%). One-hundred-eleven (62%) participants indicated that they used SOMs in their practice. The most common barriers to using the SOMs were time-consuming for patients and therapist and difficult to understand the outcome measures by the patients. Those with a Masters degree were 3.5 times more likely to use SOMs compared to PTs with diploma level qualification [Odd Ratio (95% CI) 3.5 (0.9–12.6)]. Participants with a clinical specialty were nearly 3 times more likely to use SOMs than those who do not have a specialty [Odd Ratio (95% CI) 2.9 (1.6–5.5)].

**Conclusions:**

Nearly two-thirds of the participants indicated that they used SOMs in clinical practice. Time-consuming for patient and therapist, difficult to understand the SOMs by the patients were the main perceived barriers. Years of experience, professional degree, and clinical specialty had a high probability of using SOMs. The majority of the participants showed the willingness to use SOMs in the future.

## Background

Standardized outcome measures (SOMs) are tools used for measuring the changes in the patients’ performance, function or participation over time. The national health policy has strongly recommended the routine use of outcome [1]. Valid and reliable clinical outcome measurement can support better clinical decision-making, quality assurance and/or clinical research [2]. The SOMs have been used in the research setting to evaluate the effectiveness of treatment techniques [3]. In physical therapy, a good clinical practice pattern involves regular monitoring of the health status of patients using the SOMs [4]. In addition, SOMs can provide vital information for the therapists as well as patients, and help to improve the quality of patient management [5].

With an evolving trend in health care, a more patient-focused approach has been used for reducing disability and improving the quality of life (QOL) [6]. The use of SOMs in practice will motivate the patients and improve patients’ performance in daily activities and life participation rather than traditionally measured impairments (e.g. range of motion, strength) [7].

Over the last 15–20 years, approaches in rehabilitation, in general, has been shifted from professional artistry to an evidence-based practice (EBP) [8]. The use of SOMs often has been recommended to promote EBP, as well as to improve clinical practice [9]. Along with EBP, the use of the standardized instruments for measuring patients’ activity limitation and participation restriction have been advocated by rehabilitation professionals for many years [7]. However, the incorporation of routine outcome measurement in clinical practice has been neglected to a great extent [10]. The majority of allied health professionals had reported pragmatic barriers to using the outcome measures in clinical practice [1]. A variety of facilitators and barriers to routine use of outcome measurement by allied health professionals in clinical practice were identified [1]; four major themes were classified: knowledge, education, and perceived value in the outcome measurement; practical considerations; support/priority for outcome measure use; and the patient considerations.

In the last twenty years, the use of outcome measurement in daily practice has been strongly recommended in the allied health professions [11]. However, physical therapists (PTs) are still not willing to implement the use of SOMs routinely in most of the clinical settings [7]. A recent study examined the belief of Egyptians PTs in EBP and SOMs, the extent of their adoption in clinical practice, and the perceptions of EBP and SOMs benefits and barriers to their adoption [12]. Despite more than 90% of Egyptian PTs believing in EBP and SOMs, only approximately 49% of the PTs adopted EBP and 43% adopted SOMs in clinical practice. Furthermore, in another study, approximately 90% of the participants reported that the use of SOMs improved communication between patients and therapists and it also helped to determine a plan of care [7]. However, there is a lack of information about the use of SOMs by PTs in Saudi Arabia. Moreover, no previous study had examined the current status, barriers and facilitators and perception to use SOMs by rehabilitation professionals in Saudi Arabia. Therefore, the present study intended to: (1) evaluate the extent of the use of SOMs by PTs in routine daily practice in Saudi Arabia; (2) explore the barriers, facilitators and perception in the use of SOMs during physical therapy services; and (3) examine the relationship between facility settings and the PTs characteristics and the use of SOMs.

## Methods

### Participants

The study sample comprised PTs who were working in Saudi Arabia and were commonly involved in the management of patients within different clinical setting either private or public. The PTs with an active license who were working in clinical setting were invited to participate. The PTs who were working outside of Saudi Arabia, undergraduate students, and PTs who were working in different sections like administration only or non-clinicians (not dealing with patients) were excluded from the study. The present study had ethical approval from the King Saud University Institutional Review Board (Postgraduate and Research Committee of Health Rehabilitation Sciences Department). An informed consent form was given to each potential participant to explain and obtain written acceptance. Confidentiality was maintained throughout the study.

### Procedures

The present study used an observational design. Three-hundred-fifty-two potential participants were selected from the list of PTs working in Saudi Arabia as provided by the Ministry of Health (MOH). The sample size was calculated based on an estimated 50% response rate with a 95% confidence level and a confidence interval of three or less if a response was selected by 50% of the sample. The sample was randomly selected using computer generated process and three geographic regions including Makkah, Al-Madinah Almunawrah, and Eastern province was used for stratification of the sample from the hospital outside Riyadh and rural areas. Five major public hospitals and five private hospitals were also used for stratification of the sample in the Riyadh hospitals. Based on the numbers of PTs, 20 participants for each five main public hospitals and 10 participants for each five private hospitals in the Riyadh were invited. In addition, an email with the link to electronic Google drive survey software program for a web-based survey was sent randomly to 202 physical therapists working in the hospitals outside of Riyadh and rural areas. Each potential participant received a consent form and an explanation about the aim and significance of the study as well as an overview of the survey contents and lastly an appreciation for participating in the study. A reminder was sent to the participants after three weeks if the completed questionnaire was not returned. None of the authors were known to any of the participants. Participants were known by his/her email account.

The original English version of the survey instrument was used in this study [7]. Permission was taken from the original author to use their survey tool in this present study. Cronbach alpha was calculated to determine internal consistency for each of the factors. All items related to beliefs about the facilitators and the barriers to the use of standardized outcome measures was good (α = 0.84 and α = 0.83, respectively).

### Statistical analysis

The statistical software IBM SPSS (version 21 for Windows; IBM Inc., Chicago, IL, USA) was used to analyze the data. Means and response frequencies were presented. To investigate the association of participant characteristics, including gender, working experience, qualification, specialty, and facility setting with the use of SOMs, a multivariate logistic regression analysis was conducted. Odds ratios with 95% confidence intervals were reported for each level of the independent variables. A reference group was selected for each variable for the correct interpretation of the results. A *p* < 0.05 was selected as a level of significance in all statistical analyses.

## Results

### Participants’ characteristics

From 352 invited participants, 183 responses were received (52% response rate). Two participants were excluded from the analysis because the majority of their patients were outside of the Saudi Arabia, one questionnaire was returned with no responses. The remaining 180 questionnaires were included in the analysis representing an effective response rate of 51%. The participants’ characteristics are illustrated in Table 1. Fifty-eight percent of the participants were female. The majority of the samples were baccalaureate holders (75%), and approximately 29% of participants had 6–10 years of experience. Eighty-five percent of the participants had a professional degree, and the majority (79%) was working in Riyadh. Thirty-two percent worked in an acute care setting. Approximately 52% of the sample did not carry a specialty certification. Among those who were specialized, the majority were orthopedic or neurology specialty. On average, the PTs of this current sample treated 9 patients per day.

**Table 1:** Participant characteristics (*N* = 180)

Variable	N	Percentage	Percentage of respondents that uses outcome measures
Gender			
Male	75	41.7	64.0
Female	105	58.3	60.0
Years of physical therapist practice			
< 3 years	42	23.3	50.0
3–5 years	27	15.0	74.1
6–10 years	52	28.9	73.1
11–20 years	41	22.8	60.0
> 20 years	18	10.0	39.0
Professional degree			
Diploma	21	11.7	23.8
Baccalaureate	135	75.0	63.0
Master’s	24	13.3	85.7
Specialty certification			
None	94	52.2	74.4
Orthopedic	32	17.8	
Neurology	18	10.0	
Pediatric	6	3.3	
Manual therapy	17	9.4	
Hand therapy	3	1.7	
Sports	2	1.1	
Cardiovascular	2	1.1	
Geriatric	1	0.6	
Other	5	2.8	
Type of work facility	(1 missing)		
Acute care	58	32.4	74.1
Sub-acute care	37	20.7	73.0
Extended care	37	20.7	43.2
Outpatient clinic	31	17.3	45.2
Private clinic	13	7.3	53.8
University	3	1.3	74.1
Use of standardized outcome measure			
Yes	111	61.7	
No	69	38.3	
Region			
Riyadh	142	79.0	
Makkah	25	14.0	
Al Madinah Almunawrah	5	3.0	
Eastern province	8	4.4	
Age, years (majority of patients)			
Treat all patients	25	13.9	
< 21 years	19	10.6	
21–40 years	45	25.0	
41–60 years	72	40.0	
61–75 years	18	10.0	
> 75 years	1	0.6	
Conditions			
Musculoskeletal	158	56.7	
Neurology	132	32.8	
Cardiovascular-pulmonary	60	6.4	
Women’s health	17	2.6	
Mixed	97	11.0	
Integumentary	22	2.4	
Do not manage patients	2	0.2	
		$$ \overline{X} $$	
Treatment sessions per 8-h day		9.3	

### Perceptions of standardized outcome measures

#### Perception of benefits

Of the 180 participants, 111 (62%) indicated that they used SOMs in practice. Table 2 indicates the observed benefits of using SOMs in clinical practice in the participants who used SOMs. More than 70% of the participants who used SOMs agreed that SOMs enhanced communication between the therapist and patients, increased the efficiency of examinations, helped to motivate the choice of interventions, helped to motivate and encourage patients, and to attain better patients’ outcomes.

**Table 2:** Perceived Benefits among PTs who used Standard Outcome Measures (SOMs) (*n* = 111)

Benefits	Agree		Agree somewhat		Disagree	
	N	Percent	N	Percent	N	Percent
“Helping to direct the plan of care”.	75	67.6	35	31.5	1	0.9
“Enhancing communication between therapist and patient”.	81	72.9	30	27.1	0	0
“Enhancing communication with third-party payers, physicians, and other providers”.	64	57.7	42	37.8	5	4.5
“Helping patients feel that therapists are thorough in their examination”.	74	66.7	46	41.4	1	0.9
“Increasing the efficiency of examinations”.	82	73.9	27	24.3	2	1.8
“Helping to focus choice of interventions”.	83	74.8	28	25.2	0	0
“Attaining better patient outcomes”.	79	71.2	32	28.8	0	0
“Helping to motivate and encourage patients”.	81	72.9	30	27.1	0	0
“Decreasing the rates of denial from third-party payers”.	47	42.3	56	50.5	8	7.2
“Enhanced marketing of my practice or services”.	56	50.5	40	36.1	15	13.5

### Barriers

The most common barriers to using SOMs (as shown in Table 3) were too much time taken for the therapist to calculate and analyze scores, time-consuming for patients to complete it and difficulty in completing it independently, requiring too high a reading level for many patients, confusing to patients because of the English language used in which many of the patients are not fluent. Fifty-four percent of the participants who were using SOMs reported that the use of SOMs leads to confusion for the patients. More than half of participants who used SOMs disagreed that SOMs did not contain information that helped to direct the plan of care, and culture was not a concern of our participants.

**Table 3:** Perceived barriers among PTs who used Standard Outcome Measures (SOMs) (n = 111)

Barriers	Agree		Agree somewhat		Disagree	
	N	Percent	N	Percent	N	Percent
“Confusing to patients”.	35	31.3	55	49.1	21	18.9
“Difficult for patients to complete independently”.	42	37.8	58	52.3	11	9.9
“Require too high a reading level for many patients”.	39	35.1	59	53.2	13	11.7
“English language in which many of my patients are not fluent”.	59	53.2	35	31.5	17	15.3
“Not sensitive to the cultural/ethnic concerns of many patients”.	32	28.8	53	47.7	26	23.4
“Make patients anxious”.	8	7.2	52	46.8	51	45.9
“Take too much time for patients to complete”.	53	47.7	47	42.3	11	9.9
“Take too much of clinicians’ time to analyze/calculate/score”.	48	43.2	44	39.6	19	17.1
“Provide information that is too subjective to be useful”.	21	18.9	59	53.2	31	27.9
“Require more effort than they are worth”.	20	18.1	50	45.1	41	36.9
“Do not contain information that helps to direct the plan of care”.	13	11.7	41	36.9	57	51.4
“Difficult to interpret (eg, do not know what norms are, how score relates to severity, or what a clinically important change might be)”.	14	12.6	53	47.7	44	39.6
“Do not contain the types of items or questions that are relevant for the type of patients I see”.	15	13.5	57	51.4	39	35.1
“Often do not get completed at discharge, so cannot give information about patients response to treatment”.	25	22.5	56	50.5	30	27.1

### Implementation of SOMs in practice

Approximately 60% of the participants who used SOMs reported the routine use of SOMs for examining and documenting the status, progress and/or outcomes of individual patients by an individual therapist, to communicate with the other healthcare providers, and quality improvement (as shown in Table 4).

**Table 4:** Uses of Information among PTs who used Standard Outcome Measures (SOMs) (n = 111)

Health status Questionnaires used for	Yes Routinely		Yes Sometime		No	
	N	Percent	N	Percent	N	Percent
“Answering clinical questions through a traditional research approach”.	45	40.5	62	55.9	4	3.6
“Quality improvement / assurance activities”.	59	53.2	48	43.2	4	3.6
“Determining the case mix (complexity) of patients”.	34	30.6	66	59.5	11	9.9
“Comparing performance across therapists in terms of average patient outcomes”.	49	44.1	50	45.1	12	10.8
“Comparing one clinic’s performance to that of other clinics”.	41	36.9	49	44.1	21	18.9
“Comparing average outcomes of patients with different conditions within a practice”.	45	40.5	58	52.3	8	7.2
“Examining the average change in patients’ health status over their episodes of care to determine a practice’s effectiveness”.	62	55.9	45	40.5	4	3.6
“Examining the average change in patients’ health status over their episodes of care to determine individual therapists’ effectiveness”.	55	49.5	48	43.2	8	7.2
“Examining and documenting the status, progress, and/or outcomes of individual patients by individual therapists”.	63	56.8	48	43.2	0	0
“Communicating with other health care providers and referral sources”.	59	53.2	44	39.6	8	7.20

Of the participants who used SOMs, 30% responded that they were mandated for all their patients, while 24% of them reported it was mandated only for those patients who have particular types of disorders (e.g., low back pain) and 72% used information derived from patients’ self-report. The most common way (61%) of collecting the patient’s data or analyzing the outcome was using paper followed by computer data entry by the therapists. Eighty percent of the participants used questionnaires that require patients’ self-reports completed by patients themselves, and 67% used the same health status questionnaires in their setting. The majority of participants (66%) learned how to use health status questionnaires either in professional or post- professional education level (as shown in Table 5).

**Table 5:** Organization in clinical setting

	N	Percent
“In my practice setting, completion of health status questionnaires is”,		
“Mandated/required for all patients”.	34	30.3
“Mandated only for patients who have certain types of conditions (eg, low back pain)”.	27	24.1
“Routine for all patients/clients, but not mandated/required”.	11	9.8
“Routine, but not mandated, only for patients who have certain types of conditions (eg, low back pain)”.	18	16.1
“Sporadic, depending on different factors such as time, patient’s characteristics, etc”.	22	19.6
“In my practice setting, the types of health status questionnaires used include”,		
“Only those that use information derived from patients’ self-report”.	80	72.1
“Only those that use information derived from observation of patients’ performance”.	14	12.6
“A combination of those that use patient/client self-report and observation of their performance”.	17	15.3
“In my practice setting, health status questionnaires are completed”,		
“Using paper and therapists review the raw information from the paper version”.	26	23.4
“Using paper, analyzed/scored through scanner or computer data entry, and then summary scores are reviewed by therapists”.	68	61.3
“Using the computer (no paper), and summary scores are reviewed by therapists”.	16	14.4
Other	1	0.9
“In my practice setting, when I use questionnaires that require patients self-reports”,		
“Patients complete the health status questionnaires by themselves”.	76	67.9
“Office staff or aides assist patients/clients in completing health status questionnaires”.	25	22.3
“Physical therapists complete health status questionnaires with the patients”.	10	8.9
Other	1	0.9
“Each physical therapist in my practice setting”;		
“Uses the same health status questionnaires”.	75	67.0
“Chooses the health status questionnaires he or she wants to use for each patient”.	37	33.0
“I learned how to use health status questionnaires”,		
“In my professional (entry-level) program”.	38	34.2
“In my post-professional education”.	35	31.5
“From continuing education workshops/conferences”.	27	24.3
“From the other therapists or managers in my practice setting”.	11	9.9

Only 77 (69%) participants answered the open questions and provided the list of SOMs they used in their practice; 43% of participants used Numerical Pain Rating Scale (NPRS) and Visual Analogue Scale (VAS), 35% used Functional Independence Measure (FIM), and 31% used Berg Balance Scale (BBS), 22% used Fall Risk Inventory and Oswestry Low Back Pain Disability Index (OLDI), 17% used 6-Minute walk test and Timed “Up & Go” Test, 10% used 10-M gait speed, and 5% used Rolland Morris Disability Index (RMDI) and Knee Osteoarthritis Outcome Scale (KOOS).

The most frequent reasons for choosing specific SOMs by the participants were if it was easy for the patients to understand and complete quickly. However, 50% of the participants responded that they selected SOMs when it is valid and reliable, and easy to administer by the therapists (as shown in Table 6).

**Table 6:** The reasons for selecting Standard Outcome Measures (SOMs)

^a^Reasons	N	Percentage
“Can be completed quickly”.	68	61.3
“Easy for patients to understand”.	75	67.6
“Easy for clinicians to understand/interpret meaning of scores and change in scores”.	49	44.1
“Shown to be valid and reliable”.	56	50.5
“Seem to be the most common ones used in physical therapist practice”.	38	34.2
“Useful for a variety of purposes such as research, quality assurance, patient/client evaluation”.	27	24.3
“Can be analyzed electronically (scanner, computer, etc.)”.	18	16.2
“Most appropriate for the types of conditions seen in my practice setting”.	35	31.5
“Other reason”	1	0.90
“Do not know”	2	1.80

^a^May indicated more than one criteria

### Reasons for not using SOMs

Thirty-eight percent of participants indicated that they did not use SOMs in practice, with 14% indicating that they did not plan to use it in the future, while 86% reporting willingness to use SOMs in the future. The most frequent reasons for not using SOMs selected by the participants were time-consuming for the patients completing it, as well as for the therapists to analyze or calculate the score, and difficulty for the patients to complete independently. Thirty-five percent of the participants who did not use SOMs reported that the use of SOMs required specific training for improved usage, 38% responded that they believed the usefulness of SOMs is for research purposes only (as shown in Table 7).

**Table 7:** Reasons among Participants who did not use Standard Outcome Measures (SOMs) (*n* = 69)

^a^Reasons	N	Percentage
“Confusing to patients”.	30	43.4
“Difficult for patients to complete independently”.	49	71.0
“Require too high a reading level for many patients”.	29	42.0
“English language in which many of my patients are not fluent”.	30	43.4
“Not sensitive to the cultural/ethnic concerns of many patients”.	11	15.9
“Make patients anxious”.	8	11.5
“Take too much time for patients to complete”.	52	75.7
“Take too much of clinicians’ time to analyze/calculate/score”.	35	50.7
“Provide information that is too subjective to be useful”.	16	23.2
“Require more effort than they are worth”.	17	24.6
“Do not contain information that helps to direct the plan of care”.	15	21.7
“Difficult to interpret (eg, do not know what norms are, how score relates to severity, or what a clinically important change might be)”.	11	15.9
“Do not contain the types of items or questions that are relevant for the types of patients I see”.	8	11.5
“Often do not get completed at discharge, so are not useful for determining patients’ response to treatment”.	12	17.4
“Require training that I do not have”.	24	34.8
“Cost too much”.	6	8.8
“Require a support structure that I do not have (eg, technology, staffing)”.	14	20.3
“Really only useful for research purposes”.	26	37.7
“Not relevant because my practice involves consultation, case management, or discharge planning only”.	8	11.5
Plan to implement?		
Yes	24	34.8
No	10	14.5
Maybe	35	50.7

^a^May indicated more than one reason

### Participant characteristics influencing the use of standardized outcome measures

Gender of participants had no relationship with using SOMs, while years of experience, professional degree, and clinical specialty had a high probability of using SOMs. The experience of participants was related to the likelihood of using SOMs. Compared with PTs who have had experience of 3 years or less, participants with experience of 6–10 years were 2.7 times more likely to use SOMs. The professional degree of participants was related to the likelihood of using SOMs. Compared with the baccalaureate degree holder, Master’s degree holder was 3.5 times more likely to use SOMs and the PTs with diploma qualification were less likely to use SOMs. Participants with a clinical specialty were 2.9 times more likely to use SOMs than those who do not have a specialty. The type of facility in which the participants practiced showed extended care and outpatient clinics were less likely to use SOMs than acute care [Odd Ratio (95% CI) 0.3 (0.1–0.7)] (as shown in Table 8).

**Table 8:** Odds of Using Standard Outcome Measures (SOMs) by Participant and Practice Characteristics

Factors	Odd Ratio	P- value	95% CI	
			Lower	upper
Gender				
Male	Reference	0.6		
Female	0.8		0.5	1.6
Years of experience				
< 3 years	Reference			
3–5 years	2.9	0.05	0.9	8.2
6–10 years	2.7	0.02	1.2	6.4
11–20 years	1.6	0.32	0.7	3.7
> 20 years	0.6	0.43	0.2	1.9
Professional degree				
Baccalaureate	Reference			
Diploma	0.2	0.002	0.1	0.5
Master’s	3.5	0.001	0.9	12.6
Facility				
Acute care	Reference			
Sub-acute care	0.9	0.90	0.4	2.4
Extended care	0.3	0.003	0.1	0.6
Outpatient clinic	0.3	0.008	0.1	0.7
Private clinic	0.4	0.16	0.1	1.4
Specialty				
No	Reference	0.001		
Yes	2.9		1.6	5.5

## Discussion

The majority of respondents in the present study were from Riyadh - capital city of Saudi Arabia which contains the larger main general, specialized, government and private hospitals than those in other cities. The result showed that 62% of the PTs of this sample were using SOMs in their clinical practice, consistent with previous studies [2, 7, 13]. In the Netherlands, Swinkle et al. [14] reported that 70% of respondent used at least one SOM in their daily practice. In Egypt, Elsobkey et al. [12] reported that 43% of respondents incorporate SOMs in their clinical practice, whilst Jett et al. [7] reported that 48% of responding members of the American Physical Therapy Association used SOMs. Another study reported that 53% of respondents from the Dutch physical therapy population used three of the seven recommended outcome measures as per the recommended clinical practice guidelines in the management of patient with a stroke [13]. In addition, Copland et al. [15] reported that 40% of their New Zealand respondents used back pain outcome measures.

In the present study, it was found that PTs with experiences of more than 20 years were less likely to use SOMs than younger PTs. However, the difference was not statistically significant, although PTs with experiences of 6–10 years were more likely to use SOMs. This may reflect that there were few educational workshops, professional development courses in the past compared to present and more attention paid to the concern about the use of the SOM in contemporary entry level programs. Further, Swinkel et al. [14] found that younger PTs were more likely to use outcome measures, while conversely Jette et al. [7] found participants who had been practicing for more than 20 years were much more likely to use SOMs than younger colleagues.

In the present study, the participants who had a clinical specialization showed a higher percentage of SOMs usage (74%) compared to non-specialized ones and most of them were in orthopedic or neurological settings. Abrams et al. [16] reported a 66% use of SOMs amongst PTs who treat a majority of patients with orthopedic conditions. This is consistent with 91% of PTs with a neurology specialty in UK reporting use of SOMs in their practice [17].

Consistent with the study of Copeland et al. [15], in this present study participants having a Master’s degree level qualification were much more likely to use SOMs in their clinical practice. In contrast, Elsobkey et al. [12] reported that the highest professional degree achieved by their respondents had no significant impact on whether SOMs were adopted or not.

Participants were requested to list the SOMs that they frequently used in their clinical practices. In line with the previous studies of Macdermid et al. [2] and Chapman et al. [18], the most frequently listed measures reported in this present study were NPRS and the VAS for pain assessment, and OLDI for low back pain.

It was not surprising to find that the use of SOMs in private clinics was lower than other clinical settings in the present study. However, the difference was statistically not significant. The less use of SOMs in the private clinics may be due to the insurance services in Saudi Arabia not considering the use of SOMs as one of the mandatory elements in the physical therapy assessment protocol to get physical therapy services. In addition, time prioritization in private clinics may be a factor as PTs need more time to use SOMs with their patient which may interfere with efficient patient flow. Furthermore, MacDiarmid et al. [2] found that the salaried clinicians used outcome measures more than counterparts. Vanpeppen et al. [13] reported that the PTs who were working in private physical therapy practices, showed poor adherence to clinical practice guidelines on the physical therapy management of patients with stroke. Moreover, Swinkels et al. [14] reported that the PTs in private clinics used SOMs less than nursing home PTs. Similarly, the present study showed a low percentage of SOMs used by respondents in both extended care and outpatient clinic settings, which may reflect time-constraints, since these clinics are very busy with high numbers of patients which limit the amount of time available for each patient.

In the present study, 38% of respondents did not use SOMs but most of them indicated that they intended to use SOMs in the future. The majority of participants had a positive attitude towards the use of SOMs and they agreed about the advantages of SOMs. However, PTs indicated having barriers and facilitators during their daily practice similar to what have been discussed in the literature [1, 7, 12–15]. The most important barriers perceived by the PTs who used SOMs and reasons for not using SOMs detected among participants was practical considerations, including time-consuming for both patients and the clinicians to complete the SOMs. Similarly, lack of time has been cited as the most frequent barrier selected by PTs in the previous studies [1, 7, 14–16, 19]. In addition, the other notable barrier was an inability of patients to complete SOMs independently, which was consistent with Jett et al. [7] and Simmon et al. [20]. In other professions, such as occupational therapy and speech and language therapy, the same types of barriers as those reported by the PTs are evident [1]. The English language was a major barrier of the current sample faced with their patients, especially that some SOMs are not translated to Arabic “mother language”. Similarly, Jett et al. [7] have reported language barriers for patients not fluent in English.

The opinion that the SOMs could support patients’ communication to increase the efficiency of examination and treatment, to attain better patients’ outcome and to motivate patients, were similar to the results of previous studies [1, 7, 14, 21]. The high clinicians’ perceived value of SOMs can increase the likelihood of their use in practice, whilst the lack of perceived value decreases uptake [1]. The results of the present study indicated that the most frequent use of information selected by participants were routine use of SOMs in assessing the patients status, improvement, average change in patients’ health status over time to determine a practice’s effectiveness, as well as to determine therapists’ effectiveness, to improve communication with health care providers and to improve quality of patients’ services. These reflect the participant’s high perceived value of SOMs use.

Percentage of participants who use SOMs during clinical practice was fairly acceptable because almost half of the current sample considered the use of SOMs mandated by roles of hospitals either for all patients or certain types of condition. High level of organizational commitment and facility support could facilitate increased routine use of SOMs.

In the present study, the majority of participants have learned how to use SOMs in their professional education training; because of this, it is suggested that the education providers put more focus and attention on the use of SOMs in entry-level PT programs to facilitate the routine use of SOMs in clinical practice. In addition, SOMs were viewed to be more favorable to the participants if it is easy for patients to understand, to complete it quickly and independently, easy to interpret it and should be valid and reliable. Furthermore, it is recommended to make some following changes in the organization policy in different areas to promote the use of SOMs in Saudi Arabia. First, the use of SOMs made as the mandatory elements during the assessment and treatment of patients, via policy and procedures. Second, information about SOMs should be available in the professional journals, newsletters, and guidelines. Third, technical support, training, and access to software, and how to use and interpret the results of the SOMs in daily practice should be provided. Fourth, an educational opportunity should be available for PTs to increase their knowledge and skills about the use of SOMs during entry-level training and induce some of the changes in professional behavior. Finally, allocation of more time for each session to allow patient and therapist to obtain more opportunity to integrate the use of SOMs in clinical practice.

### Limitations

The present study had some potential limitations. The present sample cannot be generalized about the total population in Saudi Arabia because the majority of them were in Riyadh city, it would not be representative enough. The results of the present study based on the participant ‘self-report data that sometimes differ from reality. In addition, using two different ways for data collection, because the electronic survey had lower responses which forced us to use another way for more participants’ recruitment, may reduce the validity of the results.

## Conclusions

Standardized outcome measures are important tools that can provide valuable information for the patient and therapist, and help guide patient management. Nearly two-third of the participants indicated that they used SOMs in their practice. Most of them perceived that their uses enhanced examination and documentation of patient progression, determination of practice’s effectiveness, communication with the patient and quality improvement. While, time-consuming for the patient and therapist, confusing and difficult to understand the SOMs were the main perceived barriers. There was a relationship between therapist characteristics and practice settings and the likelihood of using SOMs.

## Abbreviations

BBS: Berg Balance Scale
FIM: Functional Independence Measure
KOOS: Knee Osteoarthritis Outcome Scale
NPRS: Numerical Pain Rating Scale
OLDI: Oswestry Low Back Pain Disability Index
PTs: Physical Therapists
RMDI: Rolland Morris Disability Index
SOMs: Standardized Outcome Measures
VAS: Visual Analogue Scale
